# EGFR Monoclonal Antibodies in the Treatment of Squamous Cell Carcinoma of the Head and Neck: A View beyond Cetuximab

**DOI:** 10.1155/2012/901320

**Published:** 2012-09-27

**Authors:** Scott A. Kono, Missak Haigentz, Sue S. Yom, Nabil Saba

**Affiliations:** ^1^Aerodigestive and Thoracic Tumor Program, Winship Cancer Institute, Emory University, Atlanta, GA 30322, USA; ^2^Montefiore Medical Center, Albert Einstein College of Medicine, Bronx, NY 10467, USA; ^3^UCSF School of Medicine, University of California San Francisco, San Francisco, CA 94115, USA

## Abstract

Squamous cell carcinoma of the head and neck (SCCHN) is a prevalent disease both in the United States and worldwide with an overall poor prognosis, in part due to limited activity of existing therapy. Primary therapy is largely dictated by the anatomical origin of the cancer and whether distant disease is present. Many patients with localized disease are treated with chemoradiotherapy, either in the definitive or adjuvant setting, and those with metastatic disease are treated with palliative chemotherapy. The chemotherapy used in SCCHN can be toxic, whether given with radiation or alone. The epidermal growth factor receptor (EGFR) is highly expressed in SCCHN and serves as a logical therapeutic target. EGFR-directed monoclonal antibodies (MoAbs) have higher activity in SCCHN than small molecule tyrosine kinase inhibitors. Cetuximab, a widely studied EGFR MoAb, is FDA approved in the metastatic setting, as well as with radiation for locally advanced disease. Despite improvements in survival when cetuximab is incorporated with chemotherapy for metastatic disease, the prognosis of patients remains poor. Novel EGFR MoAbs are being developed with the goal of improving efficacy and tolerability. This paper will summarize the use of EGFR-directed MoAbs in treating SCCHN with a focus on novel agents being tested.

## 1. Introduction

 Approximately 52,140 individuals (37,870 men and 14,270 women) were diagnosed with cancer of the oral cavity, pharynx, and larynx in the United States in 2011, and an estimated 7,900 people died of this disease [1]. Worldwide, an estimated 263,900 diagnoses and 128,000 deaths from oral cavity cancer occurred in 2008 [2]. This paper will focus on the most common histology of malignancy arising in the head and neck, squamous cell carcinoma (SCCHN), which highly expresses the epidermal growth factor receptor (EGFR) protein. While smoking and alcohol use are known synergistic risk factors in developing this disease, the incidence and prevalence of smoking- and alcohol-unrelated, human papillomavirus- (HPV-) associated oropharyngeal cancers is increasing [3, 4]. Biologically, and clinically, HPV-related and unrelated cancers likely represent two distinct diseases, though presently these are treated in the same manner, unless on a clinical trial [5].

 Surveillance epidemiology and end results (SEER) data (2000–2008) for cancers of the oral cavity and pharynx reveal that 34.5% of patients presented with local disease, 44.2% with regional involvement, and 14.2% had distant metastases [6]. This underscores the importance of effective therapy to eradicate local disease and achieve durable local control. Dual modality treatment with chemotherapy and radiation has dramatically improved local disease control and resultant survival; however, these surviving patients continue to have a significant risk of distant failure with metastatic disease. Palliative cytotoxic chemotherapy for metastatic disease has been marginally effective at improving survival, and difficult for many patients to tolerate. The high prevalence of the EGFR protein overexpression in SCCHN makes this a logical therapeutic target, and many studies have shown EGFR monoclonal antibodies (MoAbs) to be more active than small molecule tyrosine kinase inhibitors in this disease. Here, we will review the current use of EGFR MoAb-directed therapy in SCCHN- as well as provide an overview of novel EGFR MoAbs in development.

## 2. EGFR in SCCHN

 Activation of the proto-oncogene EGFR is an early event in head and neck carcinogenesis. EGFR mRNA is highly expressed in SCCHN and contributes to the pathogenesis of this disease [7–10]. High levels of EGFR protein expression, as detected by immunohistochemistry (IHC)- have been seen in up to 90% of SCCHN tumors and is associated with poor prognosis [8, 11].

 Wheeler et al. prospectively evaluated patients surgically treated (with no EGFR-directed therapy) for SCCHN and patients treated with radiation plus cetuximab, an EGFR-targeting MoAb [12]. EGFR protein expression was elevated in both groups of patients- and correlated with elevated *EGFR* gene copy number. Consistent with EGFR expression as a poor prognostic factor, total EGFR and activated (phosphorylated) EGFR PY1068 were independently associated with decreased progression free survival (PFS). 

 Although HPV infection appears to correlate with improved prognosis in SCCHN, its relationship with EGFR expression is under investigation [13, 14]. Kumar et al. retrospectively correlated EGFR and p16 protein expression (a marker of oncogenically active HPV infection) in oropharyngeal tumors- and found that patients whose tumors expressed low EGFR and high p16 had better clinical outcomes in comparison to those whose tumors expressed high EGFR and low p16 [15]. Though EGFR is an attractive target in treating SCCHN, neither increased expression nor gene copy number has been shown to be predictive of response to EGFR-directed therapy (referenced below). Analysis of ongoing trials and prospective studies will be necessary to further define the relationship between EGFR and HPV status as it relates to treatment response and patient survival. 

## 3. Induction Chemotherapy (ICT)

 As advances in concurrent chemoradiotherapy (CRT) for locally advanced SCCHN improve local control and overall survival, distant failure has become a significant cause of mortality in this patient population, especially among patients with presenting with advanced nodal disease. Evidence exists that induction chemotherapy (ICT) may decrease distant metastatic failure; however, no clear survival advantage has been shown with the use of ICT over conventional CRT [16, 17]. In a comparison of two induction regimens in patients with SCCHN ultimately treated with CRT the addition of docetaxel to a cisplatin/5-FU induction regimen significantly improved locoregional control and overall survival [18]. 

 Whether ICT can be made more tolerable and effective by incorporating EGFR MoAb use has been the focus of investigation. Cetuximab, a chimeric IgG1 EGFR-directed MoAb, has been extensively studied in clinical trials among many tumor types. Kies et al. examined weekly ICT with carboplatin (AUC 2), paclitaxel (135 mg/m^2^), and cetuximab (400 mg/m^2^ loading, then 250 mg/m^2^ weekly), followed by either definitive radiation (RT), CRT sensitized with cisplatin (100 mg/m^2^ days 1 and 22), or surgery in 47 patients with oropharyngeal SCCHN [19]. The ICT regimen was tolerable, with the most common grade 3 or 4 toxicities being skin rash and neutropenia, and importantly all patients received their intended subsequent definitive therapy. ICT yielded a 19% complete response (CR), 77% partial response (PR), and 4% stable disease (SD) rate. Overall survival (OS) at 1 and 3 years was 95% and 91%, respectively. Of six recurrences, five included distant metastatic involvement. This phase II trial showed that the addition of cetuximab did not significantly increase toxicity or compromise the ability to deliver the intended definitive therapy. 

 In a phase II study ECOG 2303 study, Wanebo et al. treated 70 stage III/IV SCCHN patients with induction cetuximab (400 mg/m^2^ loading, then 250 mg/m^2^ weekly) plus paclitaxel (90 mg/m^2^ weekly), and carboplatin (AUC2) followed by RT sensitized with weekly cetuximab (250 mg/m^2^), paclitaxel (30 mg/m^2^), and carboplatin (AUC 1) [20]. This regimen resulted in 59% clinical CR after ICT, a PFS of 66%, 2 year OS of 82%, and 8% distant failure rate. Toxicity data were not reported, but the trial did provide further information regarding the efficacy of introducing EGFR MoAb use in an induction regimen.

 Although phase II trials of ICT have had encouraging results, no survival benefit has been shown with the use of ICT overconcurrent CRT. Results of two large trials examining ICT were recently presented at ASCO. The DeCIDE (NCT00117572) trial randomized 280 patients with high-risk SCCHN (N2/N3 disease) to RT-plus docetaxel, fluorouracil, and hydroxyurea versus two cycles of ICT (docetaxel 75 mg/m2, cisplatin 75 mg/m2, and fluorouracil 750 mg/m2 days 1–5) followed by the same CRT regimen [21]. No differences were seen in 3-year OS with a rate of 75% in the ICT arm and 73% in the CRT arm, despite a significantly lower rate of distant failure favoring the ICT arm (10% versus 19%, *P* = 0.025). The phase III PARADIGM trial evaluated induction TPF (docetaxel, cisplatin, 5-FU) × 3 followed by CRT (weekly carboplatin and daily RT, or weekly docetaxel and accelerated boost RT) versus CRT (accelerated boost with bolus cisplatin × 2) in patients with locally advanced SCCHN. Accrual was poor prompting early termination with only 145 of 300 planned patients enrolled. No difference in 3-year OS was observed—73% in the ICT group, and 78% in the CRT group. Three year PFS were similar, 67% and 73% in the ICT and CRT groups, respectively [22]. Whether EGFR MoAbs may improve efficacy and tolerability of ICT regimens remains an active area of investigation.

## 4. EGFR MoAb Use with Definitive ****Radiotherapy

 The treatment disposition of SCCHN is largely dictated by primary tumor site and stage. Surgical resection is an accepted primary therapy for SCCHN of the oral cavity, and some early-stage oropharyngeal tumors. Most patients with locally advanced (stage III-IVA/B) oropharyngeal cancers are treated with CRT [23–25]. While definitive RT in combination with cisplatin-based radiosensitization remains the current standard of care for locally advanced unresectable SCCHN or for organ preservation regimens, the toxicities of treatment—including potentially permanent ototoxicity and nephrotoxicity—can often be prohibitive [24, 26]. 

 Combining radiation with an EGFR-targeted therapy such as cetuximab is a biologically sensible approach to potentiate the effects of radiation with less toxicity than expected with a cisplatin-based CRT. Radiation therapy has been shown to increase EGFR expression in cancer cells, which potentially promotes radioresistance, further increasing its attractiveness as a therapeutic target [27, 28]. Using SCCHN cell lines, Lu et al. have recently shown that radiation induces HIF-1a expression; furthermore, experimentally increasing HIF-1a expression decreased radiosensitization with cetuximab, and blocking HIF-1a expression augmented responses to radiation with cetuximab in cetuximab-resistant cell lines [29]. 

 In a landmark trial, 213 patients with locally advanced SCCHN were treated with radiation therapy (RT) alone and 211 patients were treated with RT plus weekly cetuximab [25]. Patients treated with cetuximab plus RT had significantly superior median PFS (17.1 months versus 12.4 months, *P* = 0.006), locoregional control (24.4 months versus 14.9 months, *P* = 0.005), and OS (49 months versus 29.3 months, *P* = 0.03). The results from this study were recently updated and continue to show significant improvements in OS (49 versus 29.3 months, *P* = 0.018) and a 5-year OS of 45.6% versus 36.4% with the addition of cetuximab to radiation. Notably, the development of a grade 2 or greater rash was significantly associated with improved survival, compared with patients who developed grade 1 or no rash [30]. Unfortunately, the effect of HPV status on treatment response and survival data is unknown; however, it is of note that a majority of patients on this trial had oropharyngeal tumors and were young—two characteristics on subset analysis that were associated with improved cetuximab efficacy. The authors note that the trial was not powered to detect statistical significance in this subgroup analysis so conclusions cannot be drawn. 

 Preclinical studies in SCCHN cell lines have shown a synergistic effect of cetuximab with cisplatin, and clinical data in the metastatic setting (discussed below) confirm the activity of this combination [31]. RTOG 0522 was designed to test the hypothesis that the addition of cetuximab to cisplatin with RT in the frontline therapy of locally advanced SCCHN will improve PFS over standard cisplatin-based CRT [32]. A total of 895 patients were evaluable (447 treated with cetuximab plus cisplatin/RT and 448 treated with cisplatin/RT). With a median followup of 2.4 years, no significant difference in PFS or OS was seen between the study arms. One hypothesis for the lack of benefit to the addition of cetuximab to cisplatin plus radiation is whether HPV positivity may negatively influence the response to cetuximab or may have been a confounding factor in the control arm. 

 In a separate analysis, Young et al. evaluated 212 patients with SCCHN for HPV status via IHC analysis of p16, *EGFR *gene copy number by FISH, and EGFR protein expression by IHC. EGFR expression was positive in 87% and increased gene copy number in 20% of tumors, with both associated with worse failure-free and overall survival. p16 was positive in 43% of the overall population (57% of oropharyngeal cancers), and it was associated with improved failure-free and overall survival. Notably, only 2 of 126 (1.6%) oropharyngeal cancers were positive for both p16 and EGFR [33]. 

 Whether HPV positivity truly confers a clinically significant differential response to cetuximab versus cisplatin remains unknown and is the focus of the ongoing trial RTOG 1016. This trial is enrolling patients with locally advanced HPV-positive SCCHN to be treated with radiation therapy plus either cisplatin (100 mg/m^2^ × 2 doses) or weekly cetuximab (400 mg/m^2^ loading, then 250 mg/m^2^ weekly). The primary endpoint of this trial is to determine whether the use of cetuximab, rather than cisplatin, with radiation therapy, will result in comparable 5-year overall survival. Local and distant failure rate, toxicity data, and biomarker analysis correlated with survival will be obtained, with the comprehensive goal of answering the clinically practical question of treatment stratification based on HPV status.

## 5. EGFR MoAb Use in Recurrent/Metastatic (R/M) SCCHN

 Despite recent advances in primary therapy for head and neck cancer, locoregional recurrence is still common, and approximately 20% of patients with SCCHN will develop distant metastasis [34]. The majority of patients with unresectable recurrence will have palliative chemotherapy as their primary treatment option. 

 ECOG 5397 evaluated the use of cisplatin in combination with cetuximab for the first-line treatment of 117 patients with R/M SCCHN and found no significant difference in PFS (4.2 versus 2.7 months; cetuximab versus placebo, resp.) or OS (9.2 versus 8.0 months). Objective response rate was significantly increased with the use of cetuximab (26% versus 10%, *P* = 0.03), and in subset analysis, response rate trended toward improvement (33% versus 7%, *P* = 0.08) in patients who developed skin toxicity [35]. Single agent cetuximab was shown to have modest activity (13% response rate) in a phase II study evaluating cetuximab in 103 patients with R/M SCCHN refractory to platinum therapy [36]. 

 In the EXTREME study [37], 442 patients with R/M SCCHN were randomized to receive platinum based chemotherapy (cisplatin 100 mg/m^2^ or carboplatin AUC 5) plus 5-fluorouracil (1000 mg/m^2^/day × 4 days) every three weeks to a maximum of six cycles with or without cetuximab (400 mg/m^2^ loading dose, then 250 mg/m^2^ weekly), with the option for patients treated with cetuximab to continue maintenance cetuximab until disease progression or unacceptable toxicity. The addition of cetuximab to chemotherapy significantly improved median OS (primary endpoint) from 7.4 to 10.1 months (*P* = 0.04, HR death 0.80, 95% CI, 0.64–.99); median PFS increased from 3.3 to 5.6 months (*P* < 0.001; HR progression 0.54, 95% CI 0.43–0.67), and response rate improved from 20% to 36% (*P* < 0.001). Treatment with cetuximab was tolerable, though significantly more cases of sepsis (9 versus 1 patient, *P* = 0.02), hypomagnesemia (11 versus 3 patients, *P* = 0.05), and grade 3 skin reactions (*P* < 0.01) were seen with cetuximab. A retrospective biomarker analysis of this trial (312 of 442 patients, 71%) revealed 101 (32%) of tumors to have elevated *EGFR* gene copy number by FISH, but there was no predictive correlation between gene copy number and cetuximab efficacy [38]. While the pivotal EXTREME trial supported the integration of cetuximab into aggressive first-line treatment of R/M SCCHN in patients with good performance status, much room for improvement in patient survival exists. Given survival improvement observed with cetuximab as a single agent in colorectal cancer [39] and known modest activity of cetuximab in platinum-refractory disease [36], the sequence of incorporating cetuximab in treatment of R/M disease. Additionally, further study into predictive biomarkers, novel EGFR-targeting therapies, and strategies to combine these targeted agents with chemotherapy and alternative pathway inhibitors (e.g., FGF, PI3 K, mTOR, and others) warrant continued investigation.

## 6. Novel EGFR-Directed Monoclonal Antibodies 

### 6.1. Necitumumab (IMC-11F8)

 In contrast to cetuximab, which is chimeric (mouse/human), necitumumab is a fully human IgG1 MoAb-targeting EGFR that has the potential advantage of fewer skin toxicities and severe hypersensitivity reactions. Necitumumab blocks EGFR with high potency, with an IC50 of 1-2 nM, and exhibits comparable antitumor efficacy with that of cetuximab in preclinical models [40]. In a phase I trial of necitumumab in 60 patients (31 treated with every other week dosing, 29 treated with weekly dosing) with advanced solid malignancies, Kuenen et al. established the MTD at 800 mg after 2 patients (both in the every other week arm) experienced grade 3 headache after dosing. The most common side effects among the two arms included acneiform rash, dry skin, diarrhea, and hypokalemia. No hypersensitivity infusion reactions occurred. The *t*_1/2_ was 7 days and was similar between arms. A partial response was seen in 2 patients (melanoma and colorectal cancer), and stable disease was seen in 16 patients (8 in each arm) [41].

### 6.2. Zalutumumab (HuMax-EGFR)

 Zalutumumab is a fully human IgG1k MoAb-targeting EGFR. Preclinical work demonstrates competitive ligand-binding inhibition by EGF and TGF-*α* of EGFR that subsequently blocks receptor dimerization and activation, as well as antitumor effect via antibody-dependent cellular cytotoxicity (ADCC) [42, 43]. In a phase I/II study of 28 patients with incurable SCCHN treated with zalutumumab, Bastholt et al. found the most frequent adverse events (AEs) to be rash (16 of 28 patients), and no dose-limiting toxicity (DLT) was reached at 8 mg/kg with up to five doses being delivered. No anaphylaxis or severe hypersensitivity reactions were noted. Among the 4 mg/kg and 8 mg/kg dose levels, 7 of 11 patients had a partial response (PR—1 patient) or stable disease (SD—6 patients), and for the entire group, the objective response rate (ORR) on an intention-to-treat basis was 7.1% (2 of 28) and SD rate was 32.1% (9 of 28). Pharmacokinetic studies indicated both the 4 mg/kg and 8 mg/kg dose levels appeared to saturate compartments, and both doses were associated with rash indicating pharmacodynamic effect; therefore, a maintenance dose of at least 4 mg/kg was recommended for future trials [43]. 

 Machiels et al. conducted a randomized phase 3 trial of zalutumumab versus best supportive care (BSC) with optional methotrexate in patients with incurable SCCHN who progressed through platinum-based therapy within six months of enrollment [44]. Zalutumumab doses were individually titrated based on the appearance of skin rash. All patients received an 8 mg/kg loading dose, followed by two weekly doses of 4 mg/kg. Patients whose rash was ≤ grade I, increased their dose by 4 mg/kg every two weeks to a maximum of 16 mg/kg. If the rash was grade 2, then the same dose was continued. Treatment was held for grade 3 rash until return to ≤ grade 1. Weekly zalutumumab was continued until progressive disease or unacceptable toxicity. A total of 191 patients were enrolled into the zalutumumab group and 95 patients into the BSC group. Most (72%) of patients in the BSC arm received weekly methotrexate at the initiation of the trial, and an additional 6% began use during the trial. At a median followup of 6 months, 231 deaths had occurred, and no significant difference in overall survival was seen (5.2 versus 6.7 months in the zalutumumab and BSC groups, resp.). Progression free survival (PFS) was longer in the zalutumumab group compared with the BSC group (9.9 versus 8.4 weeks), and the HR for progression or death stratified by WHO performance status was 0.63. The most common grade 3-4 AEs were rash (21% zalutumumab versus 0% BSC), anemia (6% versus 5%), and infections (15% versus 9%). Serious adverse events (SAEs) included tumor hemorrhage (15% versus 14%), pneumonia (7% versus 3%), and dysphagia (6% versus 2%). The authors concluded that dose titration based on rash is safe. The lack of OS benefit may have been confounded by the widespread use of methotrexate in the control population, and relatively high median survival in the BSC arm. Additionally, upon progression more patients in the BSC received chemotherapy other than methotrexate compared with the zalutumumab arm, and a large number of patients in both arms received off-protocol therapy (14% in zalutumumab arm and 28% in BSC arm), further confounding the overall survival efficacy signal. The authors mention improved overall PFS benefit to be higher in patients with high versus low EGFR expression, though data was not shown. Zalutumumab is actively being investigated in numerous clinical trials (Table 1).

### 6.3. Panitumumab (ABX-EGF)

 Panitumumab is a fully human IgG2 MoAb directed against EGFR- and has been extensively studied and FDA approved in treating metastatic colorectal cancer. Panitumumab, being fully human, has less potential for severe hypersensitivity reactions than cetuximab. Being an IgG2 antibody, rather than IgG1, may limit panitumumab from inducing antitumor activity through antibody dependent cell cytotoxicity (ADCC) and NK cell activation; however, the notion that IgG2 antibodies cannot induce ADCC has recently been challenged, with Schneider-Merck et al. showing panitumumab-mediated ADCC through myeloid, rather than NK cells [45, 46]. The true clinical significance of myeloid versus NK-cell-mediated ADCC is unknown. 

 Preclinical data using xenograft models show that panitumumab has a high affinity for EGFR and blocks TGF-*α* and EGF binding and subsequent downstream signaling [47]. Kruser et al., using the SCC-1483 mouse xenograft model, revealed increased apoptosis with panitumumab monotherapy (measured by PARP expression), and the inhibitory effect on tumor growth was augmented when compared to EGFR inhibition or radiation alone [48]. This combination effect was further evaluated by Wirth et al., who conducted a phase I trial of panitumumab, carboplatin, and paclitaxel with radiation therapy (IMRT) in patients with locally advanced SCCHN [49]. Nineteen patients were enrolled in two paclitaxel dose levels (3 patients: 15 mg/m^2^, 16 patients: 30 mg/m^2^) given weekly with carboplatin (AUC 1.5). Panitumumab was dosed at 2.5 mg/kg weekly. Patients were treated to 70 Gy at the primary and gross nodal disease, and 60 and 64 Gy to low- and high-risk clinical target volumes. All patients completed therapy. Frequent toxicities included expected oral pain, xerostomia, mucositis, dermatitis, weight loss, and acneiform rash. One DLT (febrile neutropenia) occurred in the second dose level. The addition of panitumumab did not seem to significantly increase the toxicities of chemoradiation above those expected. All patients had a partial response, and the overall complete clinical response rate was 95%. At the time of publication, the median followup was 21 months, and 95% of patients remained disease free, leading the authors to conclude this is a tolerable and active regimen in locally advanced SCCHN that should be investigated further.

 The recent SPECTRUM trial (NCT00460265) randomized 657 patients to receive cisplatin/5-fluorouracil ± panitumumab [50]. The addition of panitumumab significantly improved median PFS (5.8 versus 4.6 months) but not OS (11 versus 9 months). As expected, skin toxicity was greater with the addition of panitumumab. Only 377 (57%) of patients had samples evaluable for HPV status, of which 22% were positive. A subset analysis presented at the 2011 European Multidisciplinary Cancer Congress (ECCO, ESMO, ESTRO) showed an improvement in median PFS (6.5 versus 5.1 months, *P* = 0.002) and OS (11.8 versus 8.6 months, *P* = 0.02) in the HPV negative cohort of patients with the addition of panitumumab to chemotherapy. The addition of panitumumab to chemotherapy was not associated with a significant improvement in OS or PFS in the HPV-positive subset of patients. Panitumumab remains the focus of investigation in numerous clinical trials for the treatment of SCCHN (Table 1).

### 6.4. Nimotuzumab (h-R3)

 Nimotuzumab is a humanized IgG1 MoAb-directed at EGFR that is currently approved for use outside of the United States and has demonstrated less skin toxicity than cetuximab or panitumumab. Preclinical data have shown nimotuzumab to bind EGFR with high affinity, inhibit cell proliferation, show antitumor activity in the mouse xenograft model, and enhance antitumor efficacy of radiation [51]. Crombet et al. evaluated nimotuzumab in a phase I monotherapy trial whereby 12 patients with advanced epithelial cancers received therapy at four dose levels. No evidence of severe toxicity was seen including anaphylactic or skin reactions, and the MTD was not reached [52]. They further evaluated nimotuzumab in combination with radiation therapy in 24 patients with unresectable SCCHN. Patients received six weekly doses of nimotuzumab, at four dose levels (50 mg, 100 mg, 200 mg, 400 mg), combined with RT. AEs were more frequent at higher doses with grade 1 or 2 fever, hypotension, and tremors noted to be the most common toxicities. No cases of skin rash were noted. Preliminary efficacy was a primary endpoint of the trial, and it was noted that median OS was significantly increased in the 200 or 400 mg doses (44.3 months) versus the 50 and 100 mg doses (8.6 months, *P* = 0.03). Pharmacodynamic effect was monitored with pre- and posttreatment tumor biopsies and revealed antiproliferative and antiangiogenic marker modulation by immunohistochemistry correlated with antitumor response [53]. Rodriguez et al. recently evaluated 106 treatment naïve patients with locally advanced SCCHN who were randomized to receive RT plus nimotuzumab or RT with placebo. The median OS was not significantly different (12.5 versus 9.5 months), but the CR rate (59.5 versus 34.2%, *P* = 0.038 (Fisher), *P* = 0.028 (Chisquared)) was significantly higher in the nimotuzumab plus radiotherapy group. Subgroup analysis showed a larger survival benefit with nimotuzumab in patients whose tumors expressed EGFR when compared to radiation alone (16.5 versus 7.2 months, *P* = 0.0038). Therapy was well tolerated with treatment related AEs to include fever, headache, and asthenia. No skin rash was reported [54]. 

 The use of nimotuzumab with concurrent chemoradiation, as well as in the adjuvant setting, is under investigation (Table 1).

### 6.5. ch806

 Decreased internalization and degradation of EGFR, as well as EGFR variant 3 (EGFRvIII) caused by an in-frame deletion of exons 2–7, have been associated with cetuximab resistance [55, 56]. Sok et al. analyzed EGFRvIII via IHC and quantitative PCR using 33 SCCHN tumors. They found 42% of tumors expressed EGFRvIII, and using cell lines found EGFRvIII to be associated with *in vitro* proliferation, increased tumor volume in vivo, and resistance to cetuximab (C225) therapy [56]. In a retrospective review of a phase II trial evaluating docetaxel plus cetuximab in the recurrent/metastatic SCCHN setting, Tinhofer et al. evaluated tumor biopsies from 47 patients- and found EGFRvIII expression in 17% of cases to be associated with shorter PFS and worse disease control rate, but not correlated with overall survival [57]. 

 Preclinical studies indicate that mAb-806 binds both EGFRvIII and a small proportion of wild-type EGFR, and it has shown antitumor activity in human xenograft models expressing EGFRvIII or amplified wild-type EGFR [58–61]. Scott et al. evaluated ch806 (chimeric form of mAb806) in a phase I clinical trial of eight incurable patients whose solid tumors overexpressed EGFRvIII (1 with squamous cell carcinoma of the vocal cord) and found dosing up to 40 mg/m^2^ to be well tolerated without dose-limiting toxicity. Transient pruritis, nausea, fatigue, and transaminitis were toxicities attributable to ch806. Interestingly, in biodistribution assays, significant tumor uptake of ^111^In-ch806 was seen in all patients at all dose levels, while no uptake was seen in normal tissues. After the one-month study period, 5 patients had stable disease and three had progressive disease [62].

## 7. Discussion

 Worldwide, a large number of people is affected by SCCHN. Most patients present with locoregionally advanced disease, and ongoing clinical trials seek to improve outcomes and reduce toxicity of chemoradiotherapy. Over the past decade, the EGFR has been primary focus for biologically targeted therapies in the treatment of SCCHN, with the most active treatments being monoclonal antibodies. Cetuximab, the most studied MoAb in SCCHN, is FDA approved for use as a single agent in combination with radiation therapy in the treatment of locally advanced SCCHN, and also for recurrent or metastatic disease (either as a single agent or in combination with cisplatin and 5-fluorouracil). However, several questions regarding cetuximab in these settings remain: (1) definitive randomized trials comparing cetuximab versus cisplatin as a radiosensitizer are lacking, (2) the sequencing of cetuximab with therapy (first-line with chemotherapy versus second-line single-agent) in the setting of recurrent or metastatic disease, and (3) lack of biomarker- or subset-driven trials (e.g., HPV-associated tumors) which will be critical for establishing standards of care in these specific circumstances and in optimizing outcomes. The recently presented negative results of RTOG 0522 (lack of benefit of cetuximab in combination with cisplatin-based chemoradiotherapy) are currently difficult to explain but demonstrate the need for prospective trials to answer these questions.

 The integration of EGFR MoAbs into ICT is being investigated, given their efficacy in SCCHN and lower toxicity profile than standard induction chemotherapy. To date, the efficacy data of smaller ICT trials utilizing EGFR MoAbs shows promising efficacy and tolerability; however, much larger prospective trials are needed to adopt this therapy into routine clinical practice.

 For recurrent and metastatic disease, the integration of cetuximab with platinum plus 5-fluorouracil in the EXTREME study significantly increased median PFS and OS, while the addition of panitumumab to the same chemotherapy backbone in the SPECTRUM study improved median PFS, but not OS. Interestingly, in subset analysis of the SPECTRUM study, the HPV negative patients derived benefit with prolonged median PFS and OS with the addition of panitumumab, while the PFS and OS benefit was lost in the HPV positive cohort. Whether EGFR or HPV status may account for the different results between EXTREME and SPECTRUM trials remains to be answered. Currently, we do not differentiate our treatment by HPV status in the locally advanced or metastatic setting, though this is an active area of investigation.

 The development of novel EGFR MoAbs with improved efficacy and less toxicity, including hypersensitivity reactions, could ultimately result in a positive impact on the overall tolerability of therapy and potentially survival. Ongoing investigations continue to identify multiple mechanisms of resistance to EGFR inhibition, including increased expression of vascular endothelial growth factor [63], inhibited EGFR receptor internalization and degradation [64], the expression of the EGFR variant III mutation [56], and activation or upregulation of alternative signaling pathways (e.g., Met, fibroblast growth factor, Her3) [55, 65]. Overcoming these mechanisms of resistance will be paramount to success in maintaining a sustained response to EGFR inhibition. Further studies are needed to determine the optimal use of EGFR MoAbs in the treatment of both primary and metastatic SCCHN. 

 Finally, two important factors must be considered when designing clinical trials utilizing EGFR MoAbs: (1) the negative impact on patients' quality of life, including weekly visits for treatment in many instances- and (2) the financial cost of these medications in relation to their degree of efficacy (i.e., cost per life year gained). While a cost-benefit analysis of EGFR MoAbs is beyond the scope of this manuscript, it is interesting to note the findings of an external review group (ERG) analysis of the EXTREME study. The manufacturer reported an incremental cost-effectiveness ratio (ICER) of *£*121,367 per quality-adjusted life-year (QALY) gained and an incremental cost per life-year gained of *£*92,226, far exceeding the National Institute of Health and Clinical Excellence (organization providing treatment guidance in England and Wales) range of *£*20,000–30,000 per QALY gained; therefore, the ERG concluded the addition of cetuximab to chemotherapy, as per the EXTREME study, was not cost-effective in metastatic SCCHN [66]. As therapeutic regimens are developed, and additional knowledge is gained about these promising agents, including predictive biomarkers to identify those who may have a high response rate and clinical benefit from EGFR MoAbs, progress must be made not only in improved patient outcomes but the proper selection of patients who may benefit from these novel combinations.

## Figures and Tables

**Table 1:** EGFR-targeting monoclonal antibodies (MoAb) in development for SCCHN treatment.

Agent (mechanism)	Trial description	Identifier number (status)
Necitumumab (fully human IgG1 MoAb)	Phase I trial of IMC-11F8 in patients with advanced solid tumors	NCT01088464
	Phase I trial of IMC-11F8 in patients with tumors who have not responded to standard therapy	NCT00801177

Zalutumumab (fully human IgG1k MoAb)	Phase I/II: Zalutumumab pharmacokinetics (PK) in squamous cell carcinoma of the head and neck (SCCHN)	NCT01054625
	Phase 3: DAHANCA 19: The importance of the EGFr-inhibitor zalutumumab for the outcome after curative radiotherapy for HNSCC	NCT00496652
	Phase I/II: An open, single-dose escalation study followed by a multiple dose extension of Anti-EGF receptor human MoAb (zalutumumab) in patients with recurrent or metastatic squamous cell carcinoma of the head and neck (SCCHN)	NCT00093041

Panitumumab (fully human IgG2 MoAb)	Phase II: PRISM (panitumumab regimen in second-line monotherapy of head and neck cancer)	
	Phase II: Radiotherapy plus panitumumab compared to chemoradiotherapy with unresected, locally advanced SCCHN	NCT00547157
	Phase II: Study of addition of panitumumab to chemoradiation therapy in subjects with locally advanced head and neck cancer	NCT00500760
	Phase I: Study panitumumab plus chemotherapy and induction chemotherapy in patients with locally advanced squamous cell cancer of the head and neck	NCT00513383
	Phase II: Trial of postoperative radiation, cisplatin, and panitumumab in locally advanced head and neck cancer	NCT00798655
	Phase II: PARTNER: panitumumab added to regimen for treatment of head and neck cancer evaluation of response	NCT00454779

	Phase III: Radiation therapy and cisplatin or Panitumumab in treating patients with locally advanced stage III or IV head and neck cancer	NCT00820248
	Phase II: Randomized pharmacokinetic trial of chemotherapy with or without panitumumab in patients with R/M SCCHN	NCT00756444 (Active, not recruiting)
	Phase II: Identification of gene expression signature for panitumumab sensitivity in untreated locally advanced SCCHN (TOP 0901)	NCT01305772 (Recruiting)
Nimotuzumab (humanized IgG1 MoAb)	Phase II: Nimotuzumab in combination with TPF (cisplatin, fluorouracil, docetaxel) for head and neck squamous cell carcinoma	NCT01425736 (Recruiting)
	Phase III: nimotuzumab in combination with chemoradiation for nasopharyngeal cancer	NCT01074021 (Recruiting)
	Phase II: Induction chemotherapy with nimotuzumab in locally advanced head and neck squamous cell carcinoma (HNSCC)	NCT00910117 (Completed)
	Phase II: Safety and efficacy of target therapy combined with radiotherapy to treat senile locally advanced SCCHN	NCT01393184 (Recruiting)
	Phase II: Safety and efficacy of nimotuzumab plus neoadjuvant and concurrent chemoradiotherapy to treat oropharynx and hypopharynx cancer	NCT01516996 (Recruiting)
	Phase III: Study of Post-Op adjuvant concurrent Chemo-RT with or without nimotuzumab for head and neck cancer	NCT00957086 (Recruiting)
	Phase II: Study comparing radiation therapy alone and radiation therapy and nimotuzumab for treatment of head and neck cancer	NCT01345084 (Not yet open for recruitment)
